# Molecular and functional characterization of a Rho GDP dissociation inhibitor in the filamentous fungus *Tuber borchii*

**DOI:** 10.1186/1471-2180-8-57

**Published:** 2008-04-09

**Authors:** Michele Menotta, Antonella Amicucci, Giorgio Basili, Emanuela Polidori, Vilberto Stocchi, Francisco Rivero

**Affiliations:** 1 Istituto di Chimica Biologica "G. Fornaini," Università degli Studi di Urbino "Carlo Bo," Via Saffi 2, 61029 Urbino (PU), Italy; 2 Istituto di Ricerca sull'Attività Motoria, Università degli Studi di Urbino "Carlo Bo," Via I Maggetti 26, 61029 Urbino (PU), Italy; 3 Center for Biochemistry, Medical Faculty, University of Cologne. Joseph-Stelzmann-Str. 52, 50931 Cologne, Germany; 4 The Hull York Medical School and Department of Biological Sciences, University of Hull, Hull HU6 7RX, UK

## Abstract

**Background:**

Small GTPases of the Rho family function as tightly regulated molecular switches that govern important cellular functions in eukaryotes. Several families of regulatory proteins control their activation cycle and subcellular localization. Members of the guanine nucleotide dissociation inhibitor (GDI) family sequester Rho GTPases from the plasma membrane and keep them in an inactive form.

**Results:**

We report on the characterization the RhoGDI homolog of *Tuber borchii *Vittad., an ascomycetous ectomycorrhizal fungus. The Tb*gdi *gene is present in two copies in the *T. borchii *genome. The predicted amino acid sequence shows high similarity to other known RhoGDIs. Real time PCR analyses revealed an increased expression of Tb*gdi *during the phase preparative to the symbiosis instauration, in particular after stimulation with root exudates extracts, that correlates with expression of Tb*cdc42*. In a translocation assay TbRhoGDI was able to solubilize TbCdc42 from membranes. Surprisingly, TbRhoGDI appeared not to interact with *S. cerevisiae *Cdc42, precluding the use of yeast as a surrogate model for functional studies. To study the role of TbRhoGDI we performed complementation experiments using a RhoGDI null strain of *Dictyostelium discoideum*, a model organism where the roles of Rho signaling pathways are well established. For comparison, complementation with mammalian RhoGDI1 and LyGDI was also studied in the null strain. Although interacting with Rac1 isoforms, TbRhoGDI was not able to revert the defects of the *D. discoideum *RhoGDI null strain, but displayed an additional negative effect on the cAMP-stimulated actin polymerization response.

**Conclusion:**

T. borchii expresses a functional RhoGDI homolog that appears as an important modulator of cytoskeleton reorganization during polarized apical growth that antecedes symbiosis instauration. The specificity of TbRhoGDI actions was underscored by its inability to elicit a growth defect in *S. cerevisiae *or to compensate the loss of a *D. discoideum *RhoGDI. Knowledge of the cell signaling at the basis of cytoskeleton reorganization of ectomycorrhizal fungi is essential for improvements in the production of mycorrhized plant seedlings used in timberland extension programs and fruit body production.

## Background

During the different phases of the life cycle of mycorrhizal fungi several morphological, genetic and metabolic modifications are induced in both symbiotic partners [1-4]. It is well known that morphological modifications of some fungal species are determined by deep cytoskeleton network modifications. The cytoskeleton is involved in cytoplasm distribution and reorganization, contributes to the cell shape definition and plays a key role in cell motility and mitosis in several organisms. In filamentous fungi cytoskeleton reorganization controls the continuous deposition of glycoprotein and lipid material assigned to the membrane and cell wall synthesis, making polarized apical growth possible [5,6]. Changes in cytoskeleton reorganization have been observed in symbioses between different fungal species and specific host plant species (e.g. *Suillus bovinus *vs. *Pinus sylvestris*, *Ceratobasidium cornigerum *vs. *Spiranthes sinensis*) [7,8].

Little is known regarding these molecular processes in fungi belonging to the genus *Tuber*. Investigations carried out in the last decade have led to the identification of several genes and proteins mainly involved in the hyphal membrane and cell wall development, such as chitin synthase, protein kinase C, cell cycle regulator p21 and phospholipase A2 [9-12]. Further studies performed on the interacting *in vitro *model *Tuber borchii-Tilia americana*, where the two partners exchange molecular signals without physical contact, have allowed us to identify genes involved in polarized apical growth and host tissue infection [1]. However, very limited information is available on the signaling pathways that regulate cytoskeleton reorganization during the truffle life cycle. In a previous work we showed that *T. borchii *Cdc42 is involved in the polarized growth and that it has a fundamental role in the organization of the actin cytoskeleton [13]. Cdc42, together with Rac and Rho, belongs to the Rho family of small GTP binding proteins. Rho GTPases are involved primarily in the reorganization of the actin cytoskeleton, hence in all cell processes linked to morphological modifications, such as cytokinesis, cell motility, vacuole trafficking, secretion and apoptosis in all eukaryotes, and more recently it has been shown that they are also upstream of complex signaling pathways that modulate gene expression and cell growth [14,15].

Rho GTPases function as tightly regulated molecular switches. The cycling between the GTP-bound (active) and the GDP bound (inactive) state is regulated by three classes of proteins. Guanine nucleotide exchange factors (GEFs) stimulate the GTP-GDP exchange reaction, whereas GTPase-activating proteins (GAPs) stimulate the intrinsic GTPase activity. GDP-dissociation inhibitors (GDIs) constitute an additional regulatory element. RhoGDIs were initially named after their ability to inhibit the spontaneous dissociation of bound guanine nucleotide (usually GDP) from their partner GTPases. They have been regarded mostly as housekeeping regulators that distribute Rho proteins equally to any membrane. Recent *in vitro *studies have revealed how their modularity allows them to function both in the cytoplasm and at the membrane interfaces. By interacting with RhoGDI binding proteins, by phosphorylation or upon alterations in the lipid composition of membranes they can actively contribute to the delivery of Rho proteins to specific subcellular membranes and signaling pathways [16-18]. Several proteins, named displacement factors, have been shown to interact with RhoGDIs and modulate their activity in that they have the ability, upon over-expression, to decrease the amount of soluble Rho-RhoGDI complexes and to increase the amount of GTP-bound Rho proteins [16]. Candidate displacement factors are proteins of the ezrin/radixin/moesin family, the tyrosine kinase Etk and the p75 neurotropin receptor that have been described to induce the release of RhoA from RhoGDI [17].

RhoGDI proteins are widespread among eukaryotes. Significant deepening in the functional knowledge of RhoGDIs has been achieved in mammals and in *Dictyostelium discoideum*, in many cases through generation of knockout strains. In mammals the RhoGDI family comprises three members: the ubiquitously expressed archetypal RhoGDIα (or simply RhoGDI) [19]; RhoGDIβ (or LyGDI), which has hematopoietic tissue-specific expression [20], and RhoGDIγ (or RhoGDI3), which is membrane-anchored through an amphipatic helix [21]. Disruption of GDI1, one of the two RhoGDI homologs of *D. discoideum*, has allowed detailed functional analyses at the cellular level, uncovering a central role in the regulation of Rho signaling and consequently actin reorganization [22,23]. While the role of Rho GTPases in polar growth in fungi is well established, little is known about the roles of RhoGDI proteins in this kingdom. In *S. cerevisiae *the ability of Rdi1, the only RhoGDI homolog, to remove Cdc42 from the plasma membrane is not essential for cell growth, but overexpression results in increased sequestering of Cdc42 in the cytoplasm and growth arrest [24,25]. Rdi1 is essentially a cytoplasmic protein that localizes to the plasma membrane at the tips of small-sized buds and the mother-bud neck region [26]. Recent studies showed that the interaction of Rdi1 with Cdc42 was enhanced at sites of polarized cell growth during the cell cycle, like incipient bud sites, tips and sides of small- and medium-sized buds, and the mother-bud neck region [27].

In order to improve the knowledge on the complex molecular mechanisms responsible for the morphological changes during *Tuber *life cycle, we have undertaken the characterization of the gene encoding a RhoGDI homolog in *Tuber borchii *Vittad. The Tb*gdi *gene is expressed at higher levels in the presence of the symbiotic host or root exudates. In yeast two-hybrid and translocation assays TbRhoGDI was found to interact with TbCdc42. Because *S. cerevisiae *was not suitable as a surrogate model for functional studies, the cellular role of TbRhoGDI was addressed in heterologous complementation experiments in the model organism *D. discoideum *where the roles of Rho signaling pathways are well established [28,29].

## Results

### Cloning and sequence analysis of *Tuber borchii *RhoGDI

**Figure 1:**
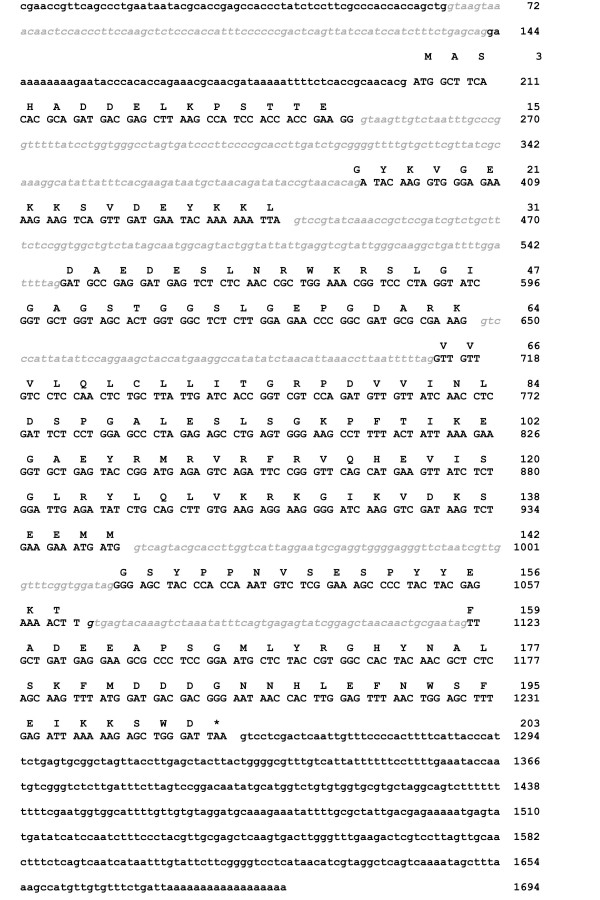
**Nucleotide and deduced amino acid sequences of the *Tuber borchiigdi *gene**. Deduced amino acid sequences are indicated on top of the nucleotide sequences. Introns are in lower case gray italics. The stop codon is marked by an asterisk. Upstream of the ATG starting codon is an adenine at position -3, in agreement with the Kozak consensus sequence. GenBank accession number EU044761.

**Figure 2:**
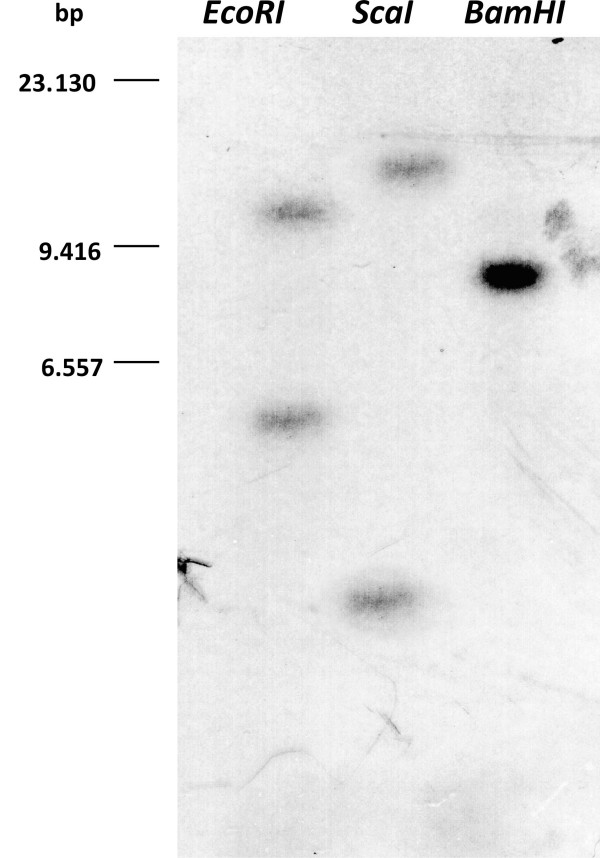
**Southern blot analysis using a Tb*gdi *specific probe**. *T. borchii *genomic DNA was digested with EcoRI, ScaI and BamHI restriction enzymes and blotted onto a nylon membrane. The blot was probed with a 207 bp long radioactively labeled probe. Tb*gdi *appears to be a two-copy gene.

A previously identified cDNA fragment encoding a RhoGDI homolog of *T. borchii *[1] was used as a probe to screen a cDNA library of *T. borchii *vegetative mycelium and 5 positive clones were isolated. The longest clone, 1365 bp in length, was entirely sequenced and shown to contain an ORF of 609 bp encoding a protein of 202 amino acids with an estimated molecular mass of 22,330 Da (Fig. 1). The gene, named Tb*gdi*, showed the highest homology with *rhogdi *of *Neosartorya fischeri*. A 315 bp long 5' untranslated region (5'-UTR), and a 3'-UTR of 441 bp were present. In Southern analyses the Tb*gdi *DNA probe detected two bands in each EcoRI and ScaI digest, suggesting that Tb*gdi *is a two-copy gene (Fig. 2). Digestion with BamHI yielded only one band, but the more intense signal leads us to conclude that the restriction fragment contains both the two copies of the Tb*gdi *gene and that the two copies lie rather close each other. Alternatively one can assume that BamHI generates two co-migrating fragments, each containing a copy of the Tb*gdi *gene, but since BamHI cuts rather infrequently in the *T. borchii *genome, the first explanation appears more convincing.

The whole Tb*gdi *genomic sequence was subsequently obtained using two pairs of primers designed at the 3' and 5' ends of Tb*gdi *cDNA and genomic DNA from mycelium as a target. The comparison between the genomic and cDNA sequences allowed us to identify five introns in the coding region, of 145, 109, 65, 69 and 57 base pairs, respectively. In addition a 78 bp long intron was detected in the 5'-UTR region. All the introns showed 5' and 3' consensus splicing sites typical of filamentous fungi [30] (Fig. 1). Upstream from the ATG start codon we identified an adenine in -3 position (3 nucleotides before the ATG codon) in agreement with the consensus Kozak sequence [31].

**Figure 3:**
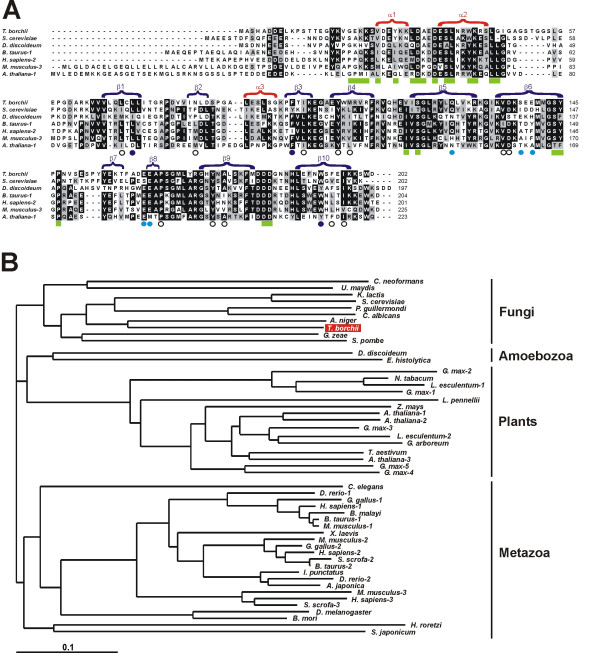
**Tuber RhoGDI and RhoGDI proteins from other organisms**. A. Multiple alignment of RhoGDI proteins from *T. borchii *and representative species. Sequences were aligned with ClustalX and the output file was subsequently edited manually. Dashes indicate gaps introduced for optimal alignment. Residues identical or similar in at least four sequences are boxed in black or grey, respectively. Secondary structure elements are indicated on top of the aligned sequences and are based on the structure of human LyGDI [61]. Residues involved in the formation of the isoprenyl-binding pocket, as determined for bovine RhoGDI [32], are indicated by open or blue circles under the aligned sequences. Blue circles indicate residues of the "hydrophobic triad" critical for binding of the distal isoprene unit. Cyan circles indicate residues involved in the formation of an acidic patch in the isoprenyl-binding pocket. Important residues involved in interactions with the Rho GTPase are indicated by green boxes and are compiled for bovine RhoGDI [32] and human LyGDI [61]. B. Phylogenetic tree showing the relationship of TbRhoGDI with RhoGDI proteins of selected species from several eukaryotic phyla. Sequences were aligned using the ClustalX program with a BLOSUM62 matrix and default settings, followed by manual edition. Only the GDI core, devoid of hypervariable amino-terminal sequences, was considered. Phylogenetic trees were constructed using the neighbor-joining algorithms of the ClustalX program. Construction of the tree was done with TreeView. The position of TbRhoGDI is highlighted. Note grouping of TbRhoGDI with other fungi, in particular ascomycetes. The scale bar indicates 10% divergence. Accession numbers of the sequences retrieved for the phylogenetic analysis are as follows. *T. borchii*, EU044761. *S. cerevisiae*, Z74183. *S. pombe*, Z98533. *C. neoformans*, EAL19587. *U. maydis*, EAK86096. *K. lactis*, CAG98029. *P. guillermondi*, EDK38281. *C. albicans*, EAL04316. *A. niger*, CAK43261. *G. zeae*, XP_385458. *H. sapiens *RhoGDI1, X69550; RhoGDI2 (LyGDI), L20688; RhoGDI-3, U82532. *M. musculus *RhoGDI1, AU080000; RhoGDI2, U73198; RhoGDI3, Q62160. *B. taurus *RhoGDI1, X52689; RhoGDI2, AF182001. *C. elegans*, U36431. *E. histolytica*, AF080396. *D. melanogaster*, AE003515. *A. japonica*, C24513. *B. malayi*, AW159949. *S. japonicum*, AAW27341. *H. roretzi*, AV383364. *I. punctatus*, BE468333. *N. tabacum*, CAB77025. *A. thaliana *RhoGDI1, AAF70843; RhoGDI2, AAC17610; RhoGDI3, AAF21198. For *S. scrofa, G. gallus, D. rerio, X. laevis, B. mori, G. max, Z. mays, T. aestivum, L. esculentum, L. pennellii *and *G. arboreum *sequences were reconstructed from diverse EST sequences.

The amino acid sequence comparison with diverse RhoGDI proteins (Fig. 3A) recognized the two functional domains characteristic of this protein family as revealed by biochemical and structural studies: a short N-terminal regulatory domain (residues 5–55) linked by residues 56–64 to a C-terminal domain with β-barrel structure (residues 65–202) [32-34]. The C-terminal domain has an immunoglobulin-like fold that contains a hydrophobic pocket for insertion of the isoprenyl moiety of the Rho GTPase. The non-polar residues that form this pocket are conserved in TbRhoGDI (Fig. 3A, open and blue circles), particularly the "hydrophobic triad" constituted by L77, F102 and W194 of bovine RhoGDI, corresponding to L72, F98 and W193 of TbRhoGDI (Fig. 3A, blue circles). This triad is critical for the formation of the binding site for the distal isoprene unit. Most residues involved in the formation of an acidic patch at the hydrophobic pocket are also present in TbRhoGDI (Fig. 3A, cyan circles). These residues interact with the polybasic region at the C-terminus of the Rho GTPases. The deduced amino acid sequence of TbRhoGDI also shows that most residues involved in interactions with Rho-GTPases (Fig. 3A, green boxes) are also conserved in TbRhoGDI.

A phylogenetic tree shows the position of TbRhoGDI in the context of a broad sample of RhoGDI proteins from other fungal species and representative members of animal, plant and amoebozoan RhoGDI proteins (Fig. 3B). From the tree it becomes evident that RhoGDIs from different phyla group in distinct clusters. TbRhoGDI clearly groups with all other fungal RhoGDIs, and is found in a cluster with sequences from ascomycetes, clearly separated from the basidiomycetes *C. neoformans *and *U. maydis*. Consequently, similarity of TbRhoGDI to fungal RhoGDIs was high (above 60%; over 45% identity), and similarity to RhoGDIs from other phyla ranged between 49% and 57% (33–38% identity).

### Expression analyses by real time PCR: a comparison between Tb*gdi *and Tb*cdc42*

**Figure 4:**
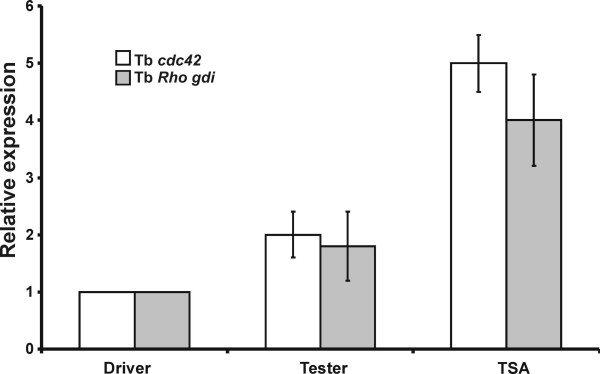
**Expression of the Tb*gdi *gene**. Real time PCR quantification of Tb*gdi *and of Tb*cdc42 *in *Tuber borchii *mycelia grown in the presence of the host plant (Tester) or root exudates (TSA) compared to untreated mycelia (Driver). The ΔΔCT method was used as described in Material and Methods. Data are average ± standard deviation of at least four independent experiments each performed in triplicate.

In order to evaluate Tb*gdi *expression in the vegetative mycelium in the absence (Driver) or presence of the symbiotic host (Tester), and in the presence of volatile organic compounds from root exudates (TSA) we set up a quantitative real time PCR assay. We designed a suitable primer pair with a high melting temperature encompassing a splice junction to prevent genomic amplification; hence non-specific products were not generated. The average Ct values for Tb*gdi *were normalized against average Ct values for the 18S rRNA. The expression differences in the three tissues were extrapolated using the ΔCT average of the Driver as a calibrator. In the presence of the plant (Tester), the fungus expressed a 1.8-fold increased amount of Tb*gdi *mRNA while the stimulated samples (TSA) showed an expression slightly below four-fold that of the control samples (Driver) (Fig. 4). The differences among the ΔCT medians of the analyzed samples were statistically significant (Kruskal-Wallis test). These expression results were compared with those previously reported for Tb*cdc42 *[13], in order to highlight the tight link between the two proteins. For both genes a higher expression in the presence of the plant and of root exudates was observed, although the increase of Tb*cdc42 *in the TSA samples was more pronounced than that of Tb*gdi*.

### Interaction of TbRhoGDI with Rho GTPases

**Figure 5:**
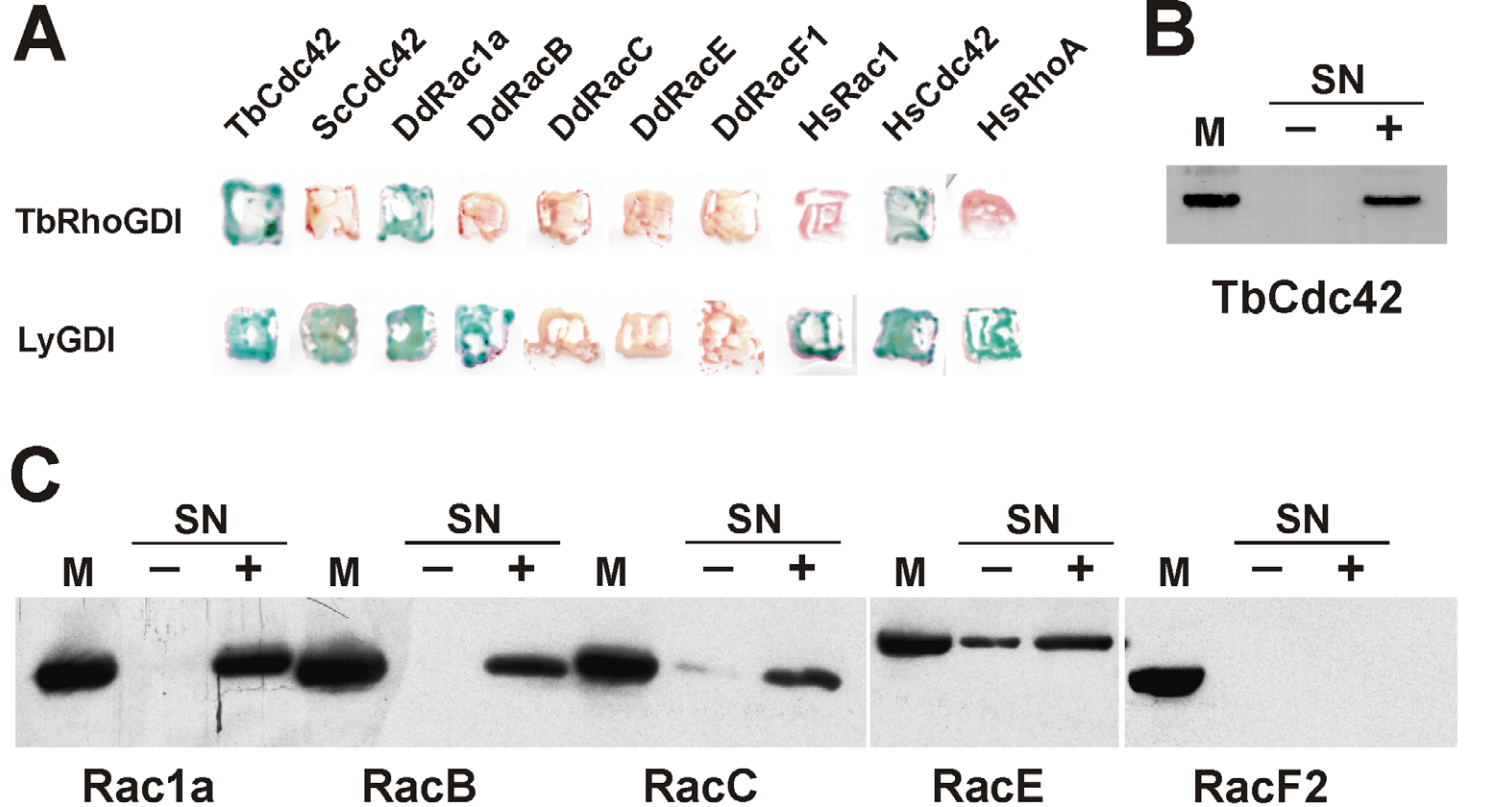
**Interaction of RhoGDIs with Rho GTPases**. A. Two-hybrid interactions between TbRhoGDI or LyGDI and the indicated Rho GTPases. After co-transformation of the corresponding plasmids into the Y190 yeast strain, colonies were allowed to grow on -Trp/-Leu plates and interactions verified and in colony-lift β-galactosidase assays. DdRac1b and DdRac1c behaved like DdRac1a (not shown). B. Translocation of TbCdc42 by TbRhoGDI. A membrane fraction of HeLa cells expressing GFP-tagged TbCdc42 was incubated in the absence (-) or presence (+) of 40 μM purified bacterially expressed His-tagged TbRhoGDI. The membranes were sedimented by centrifugation and aliquots of the supernatants were subjected to SDS-PAGE. The aliquot of membrane fraction (M) corresponds to the percentage of the analyzed supernatant (SN). TbCdc42 was detected with an antibody reactive against GFP. In these experiments His-tagged TbRhoGDI was always recovered in the supernatant. C. Translocation of *D. discoideum *Rho GTPases by bovine RhoGDI1. Membrane fractions of insect cells infected with baculoviruses encoding the indicated *Dictyostelium *GST-tagged Rho GTPases were incubated in the absence (-) or presence (+) of 40 μM purified bacterially expressed His-tagged RhoGDI1. The membranes were processed as in B. GTPases were detected with an antibody reactive against GST. In these experiments His-tagged RhoGDI1 was always recovered in the supernatant.

To date only one Cdc42 homolog has been described in *T. borchii *[13]. We therefore investigated whether TbRhoGDI is able to interact with TbCdc42 using a yeast two-hybrid approach. For reasons that are explained below, we extended our analysis to several RhoGTPases of *D. discoideum *(Rac1a, Rac1b, Rac1c, RacB, RacC, RacE and RacF1) and human (Rac1, Cdc42, RhoA) as well as to *S. cerevisiae *Cdc42 (Fig. 5A and Table 1). We observed a strong interaction of TbRhoGDI with TbCdc42. Additionally, TbRhoGDI interacted with human Cdc42, but not with Rac1 or RhoA. Surprisingly, TbRhoGDI did not show interaction with *S. cerevisiae *Cdc42 in the yeast two-hybrid analysis. When *Dictyostelium *Rho GTPses were examined, we observed good interaction with Rac1 isoforms, but not with several other Rho GTPases. We verified the interaction of TbRhoGDI with TbCdc42 using a translocation assay with recombinant His-tagged TbRhoGDI (Fig. 5B). This assay tests the ability of a RhoGDI to solubilize a prenylated Rho GTPase from a membrane preparation, an activity that is characteristic of RhoGDIs.

**Table 1:** Summary of interactions of RhoGDIs with Rho GTPases. Interaction of TbRhoGDI and LyGDI with Rho GTPases was determined using a yeast two-hybrid assay (Fig. 5A). Interaction of bovine RhoGDI with the indicated Rho GTPases was determined in translocation experiments (Fig. 5C). Data on *Dictyostelium *RhoGDI1 was taken from Rivero et al. [23], except for the interactions with TbCdc42 and ScCdc42. DdRacF1 was used in yeast two-hybrid experiments, whereas DdRacF2 was used in translocation experiments. NA, not assayed.

	TbRhoGDI	RhoGDI	LyGDI	DdRhoGDI1
TbCdc42	+	NA	+	+
ScCdc42	-	NA	+	+
DdRac1a	+	+	+	+
DdRac1b	+	NA	+	+
DdRac1c	+	NA	+	+
DdRacB	-	+	+	+
DdRacC	-	+	-	+
DdRacE	-	+	-	+
DdRacF1 or F2	-	-	-	-
HsRac1	-	+	+	+
HsRhoA	-	+	+	+
HsCdc42	+	+	+	+

### Complementation of a *Dictyostelium *RhoGDI null strain with diverse RhoGDIs

**Figure 6:**
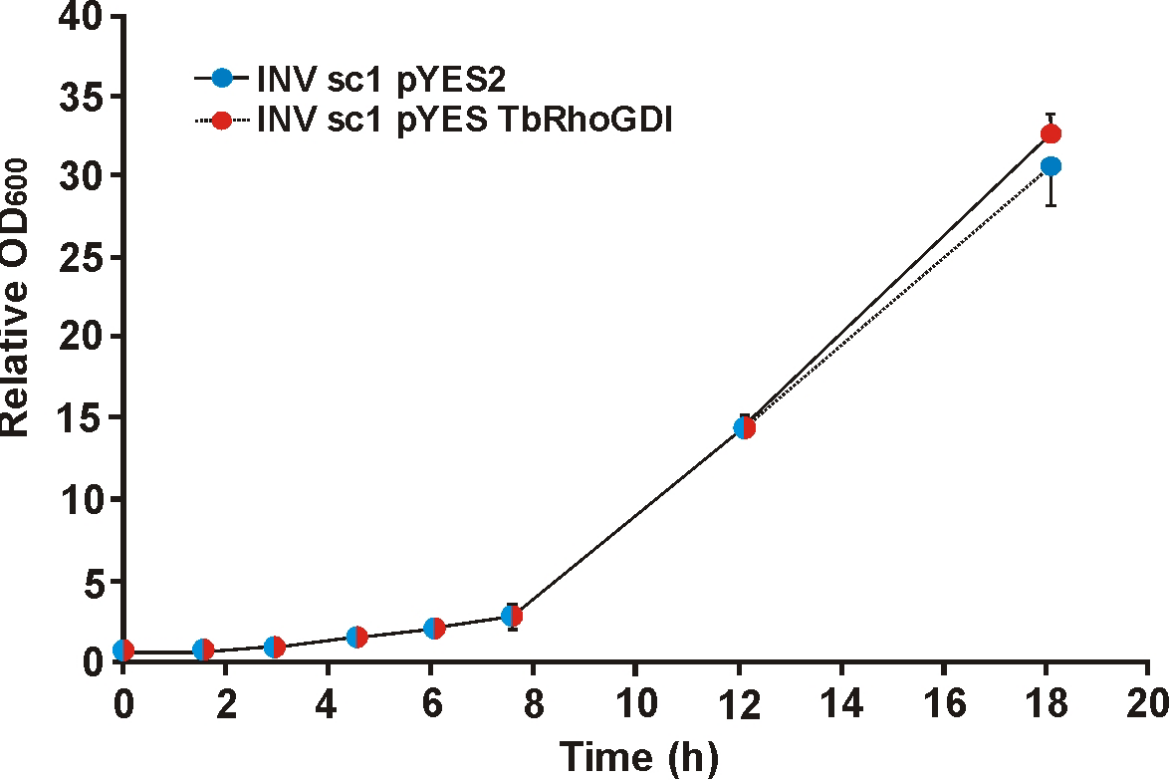
**TbRhoGDI does not alter the growth rate of *S. cerevisiae***. Yeast cells were transformed with pYES2-Tb*gdi *for expression of TbRhoGDI or with the empty plasmid as control. The OD_600 _was measured at the indicated times and normalized over the starting OD_600 _of the respective culture. Data are average ± standard deviation of three independent determinations, each done in duplicate. TbRhoGDI does not seem to interfere with the Rho signaling pathway of yeast.

The slow growth of *T. borchii *and the paucity of genetics tools available make studies at the molecular level very difficult in this organism. The absence of interaction of TbRhoGDI with yeast Cdc42 in spite of the overall higher similarity of TbRhoGDI to other fungal orthologs precluded the use of *S. cerevisiae *for functional studies. When overexpressed, the yeast RhoGDI brings about a reduction in the growth rate, and the same effect has been reported when mammalian RhoGDI is introduced into yeast [24]. In agreement with the yeast two-hybrid data, we observed that TbRhoGDI is unable to cause growth inhibition when expressed in *S. cerevisiae *(Fig. 6).

In order to gain insight into the role of TbRhoGDI, we switched to the genetically tractable model organism *Dictyostelium discoideum*. In this organism deletion of the gene encoding a typical RhoGDI results in low growth and accumulation of multinucleate cells as well as in an altered F-actin polymerization response upon cAMP stimulation. These defects can be reverted by re-expression of the ablated protein [23]. We used this GDI1^- ^strain to investigate whether expression of TbRhoGDI could restore the defects observed in these cells. For comparison, we included in this study two mammalian RhoGDI proteins, bovine RhoGDI and human LyGDI.

We first investigated the ability of the two mammalian RhoGDI proteins to interact with *D. discoideum *RhoGTPases in the two-hybrid assay. In these experiments LyGDI was found to interact with Rac1a/1b/1c and RacB (Fig. 5A and Table 1). Our attempts to use bovine RhoGDI as prey for the two-hybrid analysis were not successful, presumably because this protein interferes with the Rho signaling system of yeast [24]. We therefore used translocation assays with purified bacterially expressed His-tagged RhoGDI and observed clear interaction with Rac1a and RacB, weak interaction with RacE and no interaction with RacF2, a pattern similar to that of *D. discoideum *GDI1 (Fig. 5C and Table 1).

**Figure 7:**
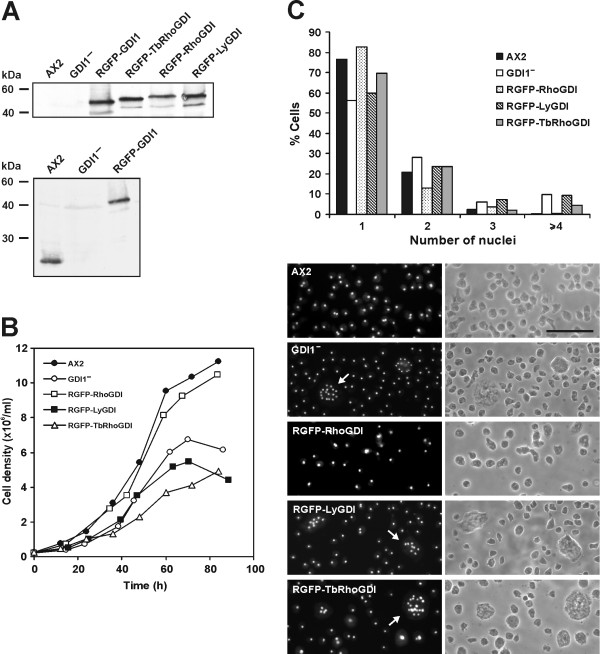
**Complementation of a *Dictyostelium *RhoGDI1 null mutant with *T. borchii *and mammalian RhoGDIs**. GDI1 null cells were transfected with plasmids that allow expression (indicated by an R, for rescue) of GFP fusions of *Dictyostelium *RhoGDI1(GDI1), TbRhoGDI, bovine RhoGDI1 and human RhoGDI2 (LyGDI). The wild-type *Dictyostelium *strain AX2 was used as reference. A. Total cell homogenates of 4 × 10^5 ^cells were resolved in 12% polyacrylamide gels and blotted onto nitrocellulose. The blot of the upper panel was incubated with a GFP-specific mAb K3-184-2. The blot of the lower panel was incubated with *Dictyostelium *GDI1-specific mAb K8-322-2. All GFP fusions are expressed at levels comparable to those of the endogenous *Dictyostelium *GDI1. B. Growth of GDI1^- ^and complementation mutants in shaking suspension. GDI1^- ^mutant cells have a reduced growth rate and reach lower cell densities than the wild type. This defect was restored after expression of bovine RhoGDI1, but not by TbRhoGDI or human LyGDI. Curves are representative of at least three independent determinations, each done in duplicate. C. Distribution of the number of nuclei in GDI1^- ^and complementation mutants. Cells were allowed to grow on coverslips, then fixed with cold methanol and stained with DAPI. 300 cells of each population were scored. Photographs show DAPI staining (left panels) and phase contrast (right panels). Arrows point at examples of giant multinucleate cells. Scale bar, 50 μm. In the GDI1^- ^mutant, cells with four or more nuclei account for about 10% of the population. This defect was restored completely after expression of bovine RhoGDI1 and partially after expression of TbRhoGDI, whereas human LyGDI had no effect.

We then introduced GFP fusions of TbRhoGDI, RhoGDI and LyGDI into the GDI1^- ^strain. Each rescue strain expressed the corresponding fusion protein of the expected size. Expression levels were comparable to those of the strain re-expressing *D. discoideum *GDI1, which in turn were similar to the endogenous levels (Fig. 7A). Expression of bovine RhoGDI (RGFP-RhoGDI strain) resulted in a complete reversion of the growth and cytokinesis defects characteristic of GDI1^- ^cells (Fig. 7B,C). By contrast, expression of TbRhoGDI or LyGDI (strains RGFP-TbGDI and RGFP-LyGDI, respectively) did not revert the growth defect (Fig. 7B). The cytokinesis defect was restored only partially after expression of TbRhoGDI, whereas LyGDI had no effect (Fig. 7C). The failure of TbRhoGDI and LyGDI to restore the growth and cytokinesis defects cannot be explained by non-uniform expression levels of the fusion proteins in the cell populations because we observed giant multinucleate cells displaying higher than average fluorescence levels.

**Figure 8:**
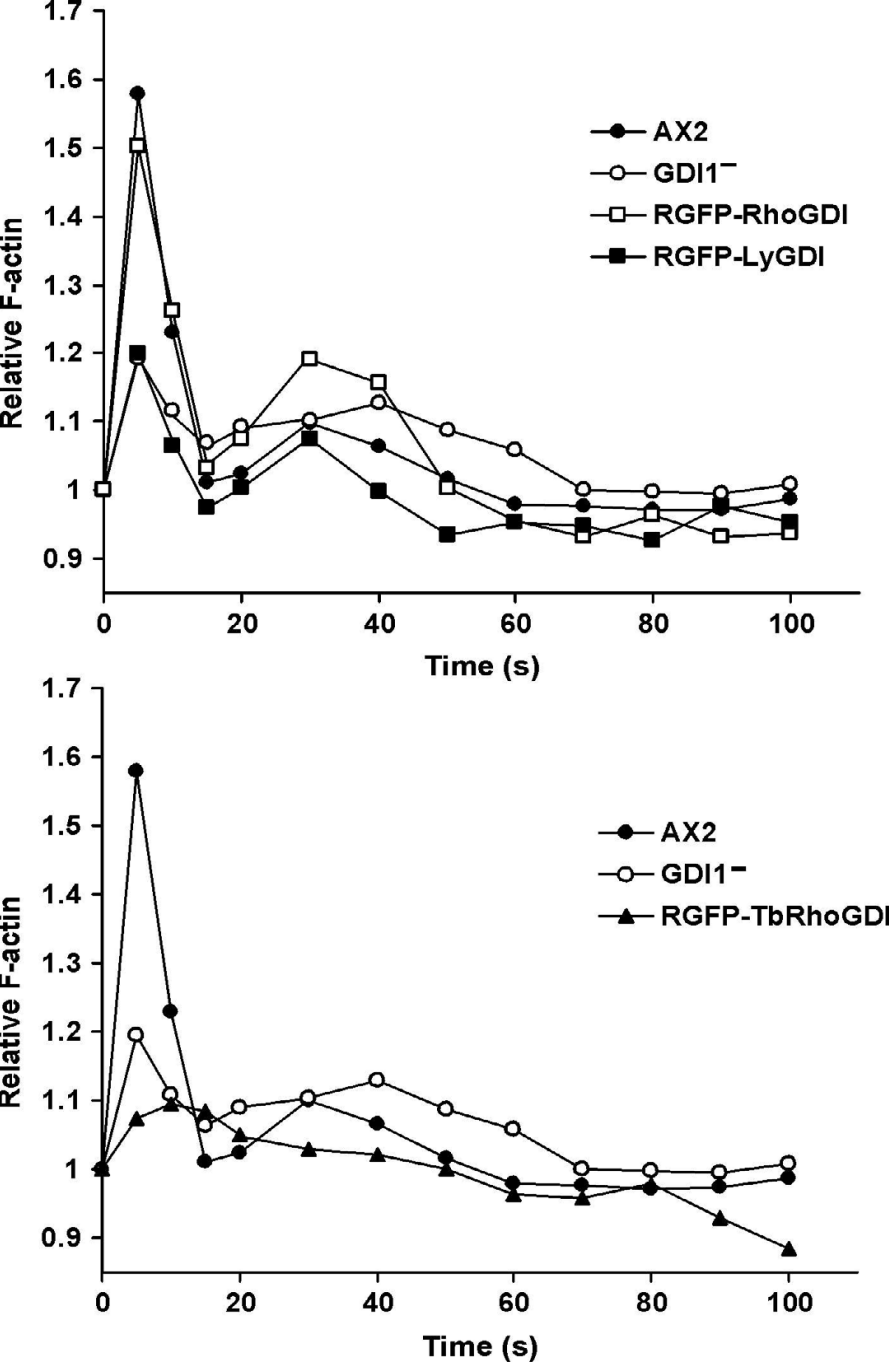
**Actin polymerization response in *Dictyostelium *upon cAMP stimulation of aggregation competent cells**. The relative F-actin content was determined by TRITC-phalloidin staining of cells fixed at the indicated time points after stimulation with 1 μM cAMP. Each data point represents the average of at least three independent measurements. For the sake of clarity, error bars are not shown and the results are presented in two graphs. In GDI1^-^cells the initial response of actin polymerization and depolymerization was reduced, and was restored after expression (indicated by an R, for rescue) of bovine RhoGDI1, but not human LyGDI or TbGDI. Expression of TbRhoGDI resulted in a further decreased first peak and an abolished second peak of actin polymerization.

Upon cAMP stimulation fast and highly transient changes in the F-actin content take place in *D. discoideum *cells that correlate with changes in cell behaviour [35]. Five seconds after stimulation a 1.6-fold increase in the amount of F-actin was observed in the wild type strain that decreased rapidly to basal levels after 20 seconds. A second much lower peak followed immediately and lasted until approximately 60 seconds. This response is in part dependent on RhoGDI, because GDI1^- ^cells showed a significantly lower increase in the first F-actin peak (1.2-fold), whereas the second peak was not altered (Fig. 8). Again, expression of bovine RhoGDI, but not of TbRhoGDI or LyGDI resulted in a reversion of the actin polymerization response. Interestingly, TbRhoGDI had a negative effect in the actin polymerization response of the GDI^- ^cells, with a reduced and slightly prolonged first peak and an abolished second peak (Fig. 8, lower panel).

## Discussion

In this study we focused on the role of RhoGDI during the pre-symbiotic phase and its interaction with the small GTPase Cdc42. In this phase, with the culture system used here, the fungal hyphae grow gathering along the roots and start branching, suggesting that the mycelium is able to respond to molecular messengers secreted by the plant. Among the messenger signal molecules, volatile organic compounds secreted by the host plant roots may play a fundamental role, as showed by gene expression analyses performed on TSA samples [1,36]. We hypothesize that the molecules present in the root exudates activate chemotactic and morphogenetic processes in fungal hyphae. In analogy to what happens in pathogenic fungi during an infection event [37-39], communication events with its host plants can occur also in *T. borchii*, leading in both partners to a coordinated program of gene expression, probably activated by a MAP kinase cascade controlled by Cdc42. In fact, Cdc42, recently characterized also in *T. borchii *[13] has, like other Rho-like proteins, a key role in signal transduction pathways that regulate gene expression and consequently represents one of the main modulators of cytoskeleton reorganization during polar growth [40-43].

Gene expression analyses showed that Tb*gdi *expresses almost two-fold as much when the host plant is present and four-fold when root exudates are added to the medium, and this behavior correlated with that of Tb*cdc42*. An increased expression of both genes in the presence of the host plant and/or of root exudates induces the fungus to prepare itself to the development of mycorrhizal symbiosis, making it to get physically closer to the plant roots through the prime of branching events and polar growth. The similar pattern of expression of both genes might reflect their strict functional link: the small GTPase TbCdc42 is modulated by TbRhoGDI, which would extract TbCdc42 from the cell membranes and sequester it in the cytoplasm. An increase of mycelium growing and branching during the pre-symbiosis phase is supported also by the evidence that several genes differentially expressed in this step, like syntaxin binding protein, aspartic protease and centractin-like protein [1], are involved in vescicular transport and cell wall construction, events indispensable for cell growing.

RhoGDI proteins are fairly conserved among eukaryotes. The high degree of conservation and the numerous crystallographic structures available in the Swiss-Prot database allowed to deduce the virtual three-dimensional structure of TbRhoGDI and verify the three-dimensional shape of the predicted domains (data not shown). The predicted three-dimensional structure revealed the presence of active sites matching those of RhoGDI in other eukaryotes, mainly fungi. The slight amino acidic differences observed are unlikely to cause any gross functional changes, but might explain the restricted pattern of interactions with Rho GTPases observed in this study. We hypothesized that TbRhoGDI may have the same functions that it has in other organisms, more specifically fungi. To date functional studies are difficult in *Tuber *cells due to the slow growth and the absence of tools to generate genetically modified strains. The experience gathered from previous studies on *Agrobacterium tumefaciens*-mediated transformation of ectomycorrhizal basidiomycetes such as *Suillus bovinus *[44,45] and *Hebeloma cylindrosporum *[46] has only recently been applied to *Tuber *[47], but unfortunately this technology is not yet widespread. To address the role of TbRhoGDI we have made use of genetically tractable model organisms in which Rho signaling is well studied: *D. discoideum *[28] and *S. cerevisiae *[48]. As revealed in the yeast two-hybrid experiments, it appears that TbRhoGDI is tailored to interact preferentially with Cdc42 but displays an exquisite specificity, as it did not interact with the yeast ortholog, thus precluding the use of this organism for further studies.

*D. discoideum *belongs to the amoebozoa and undergoes a characteristic life cycle that alternates a single cell stage as free living amoeba with a multicellular stage that ends with the formation of a fruiting body. The formation of the multicellular structure requires the establishment of cell polarity and the migration towards an aggregation center, a process in which Rho signaling plays a key role in orchestrating the remodeling of the cytoskeleton [49]. Taking advantage of the genetic tractability of *D. discoideum*, we performed heterologous complementation experiments. TbRhoGDI, bovine RhoGDI1 and human LyGDI were introduced successfully in a *D. discoideum *strain lacking GDI1. We observed that only bovine RhoGDI1, which displays a nearly identical pattern of specificity towards Rho GTPases like the *D. discoideum *homolog, was able to fully revert the cytokinesis, growth and actin polymerization defects of the GDI1^- ^strain. Interestingly, LyGDI, despite of having a spectrum of interactions not very divergent from that of RhoGDI1, failed to compensate the functions of the *D. discoideum *GDI1. A possible explanation for this failure is the lower affinity for binding to Cdc42 and probably other mammalian Rho GTPases reported for LyGDI [50] that might extend to the *Dictyostelium *Rho GTPases. In agreement with the restricted pattern of interaction with the set of Rho GTPases from *D. discoideum*, TbRhoGDI was unable to revert any of the phenotypes of the GDI1^- ^strain. Leaving aside the possibility that TbRhoGDI does not display the sufficient affinity for *Dictyostelium *Rac1, the missing interaction with RacB could also contribute to the absent compensation, as RacB is also required for an efficient actin polymerization response [51]. Interestingly, TbRhoGDI had a negative effect on the actin polymerization response, which can mean that still TbRhoGDI is able to interact with other signaling components apart from Rho GTPases. This observation is in accord with the recent recognition of several binding partners that modulate the activity of RhoGDI, the so-called displacement factors [16,18], that could be sequestered by TbRhoGDI resulting in altered signaling to the cytoskeleton.

## Conclusion

*T. borchii *expresses a functional RhoGDI homolog that appears to be tailored to interact specifically with TbCdc42. The specificity of TbRhoGDI actions was underscored by its inability to elicit a growth defect in *S. cerevisiae *or to compensate the loss of a *D. discoideum *RhoGDI. Our results represent a starting point to understand, yet in the pre-infection phase, the mechanisms that lead to the development of ectomycorrhiza in *T. borchii*. Further studies will allow better understanding the role of TbRhoGDI in the organization of the complex network of events underlying symbiosis instauration. The future availability of *Tuber *knock out strains might improve our knowledge of the cell signaling at the basis of cytoskeleton reorganization of this ectomycorrhizal fungus, which might in turn lead to improvements in the production of mycorrhized plant seedlings used in timberland extension programs and fruit body production.

## Methods

### Strains and culture conditions

The *T. borchii *culture consisted of vegetative mycelia of strain 10 RA grown on nylon membranes laid on solid MS/2 medium pH 6.5 with 10 g/l of glucose as a carbon source [52]. The culture was grown for 30 days at 24°C under 16-h light provided by cool white fluorescent lamps (Driver sample). The Tester sample was prepared as follows: *T. borchii *mycelia were grown under the above-mentioned conditions, but in the presence of the host plant *T. americana*, from which they were separated by a nylon membrane. The TSA (Air Stimulated Tester) samples were obtained treating *T. borchii *mycelia with *T. americana *root. Purified exudates were used to fill 20-μl capillary tubes subsequently placed on Driver sample medium for 5 days in order to allow diffusion of volatile organic compounds in the culture dish.

*Dictyostelium discoideum *wild type (AX2) and RhoGDI1 null (GDI1^-^) mutant strains [23] were grown either in liquid nutrient medium at 21°C with shaking at 160 rpm or on SM agar plates with *Klebsiella aerogenes *[53].

The *Saccharomyces cerevisiae *strain INVsc1 (MATa, *his3Δ1*, *leu2 trp1-289 ura3-52*/MATα *leu2*, *trp1-289 ura3-52*) was supplied by Invitrogen Life Technology (San Diego, California) and was maintained and cultivated using standard procedures.

HeLa cells were cultivated in DMEM medium using standard procedures.

### DNA and RNA isolation

Genomic DNA was isolated from 1-month-old cultures of *T. borchii *mycelia following the protocol described by Erland et al. [54]. Total RNA was extracted from mycelial samples using a Qiagen RNeasy kit (Qiagen, Hilden, Germany) according to the manufacturer's instructions. A DNase (Ambion, Austin, Texas) digestion step was performed before all subsequent reactions.

### Cloning of the Tb*gdi *gene

A 643 bp long DNA fragment harboring the Tb*gdi *gene was obtained as described previously [1]. It was inserted in pGEM vector II (Promega, Madison, Wisconsin) and transformed into *E. coli *XL1-Blue. After verification by sequencing, a specific pair of primers was designed and a 207 bp long PCR product was used as a homologous probe for the screening of 3 × 10^5 ^λ ZAP clones of a *T. borchii *mycelium cDNA library [55]. Library screening, subcloning and routine procedures were performed using standard protocols [56]. DNA sequences were analyzed by SeqPup ver 0.9e running on SUN JAVA VM ver 1.4.1_02. Database searches of the DNA/protein sequences were performed with the BLAST programs (NCBI).

### Southern analysis

Genomic DNA samples for Southern blot analysis (10 μg each) were digested with the restriction enzymes EcoRI, ScaI and BamHI that do not cut the probe used, and were electrophoresed on a 0.8% agarose gel. The DNA samples were blotted onto positively charged Hybond N+ nylon membranes (Amersham, Buckinghamshire, UK) according to the manufacturer's instructions, and hybridized at 65°C over night in phosphate buffer with the 207 bp Tb*gdi *fragment, which was labeled with [α-^32^P]CTP using the RediPrime labeling kit (Amersham, Buckinghamshire, UK). The final post-hybridization wash was carried out in 15 mM NaCl, 1.5 mM trisodium citrate (0.1 × SSC) and 0.1% SDS at 65°C.

### cDNA synthesis and quantitative real time PCR (qRT-PCR)

One-microgram aliquots of total RNA from Driver, Tester and TSA mycelial samples were denatured at 70°C for 2 min, and then reverse transcribed in a 10-μl reaction mixture using random hexamers as primers and the MMLV Power-Script Reverse Transcriptase from Clontech (Mountain View, California). The cDNA was then diluted 1/30 for the subsequently PCR reaction. 18S rRNA gene from *T. borchii *was used as a reference (18S RT F 5'-TGGTCCGGTCGGATCTT-3', 18S RT R 5'-CATTACGGCGGTCCTAGAAA-3'). Specific primers for Tb*gdi *(GDI RT F2 5'-ATCCACCACCGAAGGATACA-3', GDI RT R2 5'-TCTCCAAGAGAGCCACCAGT-3') were designed to amplify under the same cycling conditions (95°C for 10 min, followed by 50 cycles of 95°C for 30 sec and 60°C for 30 sec), generating a product of 141 bp comparable to the real time RT-PCR analyses performed with Tb*cdc42 *specific primers [13].

The PCR was performed in a Bio-Rad iCycler iQ Multi-Color Real Time PCR Detection System (Biorad, Hercules, California). Each 25-μl reaction consisted of 1 μl diluted cDNA, 12.5 μl of 2× Quantitect SYBR Green PCR kit and 300 nM of primers. The specificity of the amplification products was confirmed by examining thermal denaturation plots and by sample separation in a 2% DNA agarose gel. In the experiments we characterized the performance of the primers over a range of Tester template concentrations, from 1:5 to 1:500 dilutions of the starting diluted cDNA. The PCR reaction efficiency was 100.2% for the inner standard 18S (18S RT F-18S RT R primers) and 94.3% for the GDI RT F2-GDI RT R2 primers. The relative PCR efficiencies of target and reference were calculated (Applied Biosystems, user bulletin 2) and were found comparable for up to 36 PCR cycles. The amount of the target transcript was related to that of the reference gene by the ΔΔCt method (ΔCt = average Ct_target_-average Ct_*Tb18S*_) as described by Winer et al. [57]. The Kruskal-Wallis test was applied to samples obtained from at least four independent experiments. Results were considered significant if p values were <0.05.

### Yeast two-hybrid assays

Two-hybrid assays were performed using plasmids and following the protocols of the Matchmaker two-hybrid system from Clontech (Mountain View, California). A cDNA fragment encoding Tb*gdi *was inserted in the plasmid pGADT7, while Tb*cdc42 and S. cerevisiae cdc42 *cDNAs were inserted in the plasmid pGBKT7. Tb*gdi *cDNA was amplified using the GDI-ATG EcoRI 5'-CGAATTCATGGCTTCACACGCAGATGA-3' and the GDI-STOP BamHI 5'-CGGATCCTTAATCCCAGCTCTTTTTAA-3' primers with *Pfu ultra *polymerase (Stratagene, La Jolla, California) according to the manufacturer's instructions. The PCR product was cloned into pGADT7 vector previously digested with EcoRI/BamHI. pAS2-1 vectors harboring *D. discoideum *and human Rho GTPases have been described previously [23]. DNA fragments encoding bovine RhoGDI and human LyGDI were obtained by PCR using primers designed to introduce suitable restriction sites for cloning into the pACT2 vector. Plasmids were introduced into Y190 yeast cells. Colonies were tested for growth on -Trp/-Leu/-His/+3AT and in colony-lift β-galactosidase assays.

### Solubilization of membrane-bound recombinant Rho GTPases with soluble RhoGDI

Production of *Dictyostelium *GST-tagged Rho GTPases and of untagged human Rac1, Cdc42 and RhoA in insect cells has been described previously [23,58]. For production of GFP-tagged TbCdc42 in HeLa cells a plasmid was constructed subcloning Tb*cdc42 *from pGBKT7 where GFP cDNA was previously inserted in frame using NcoI and EcoRI. The fusion was excised with NcoI and BamHI and ligated blunt end in p3xFLAG-CMVT-14 (Sigma, St. Luis, MO, USA). HeLa cells were transiently transfected using Escort IV transfection reagent (Sigma, St. Luis, MO, USA) and membranes were prepared 48 h later as previously described [58]. Preparation of membranes containing recombinant GTPases from baculovirus-infected insect cells and purification of GST and GST-RhoGDI from *E. coli *have been described previously [58]. PCR products encoding bovine RhoGDI or TbRhoGDI were cloned into expression vector pQE30 or pET28+, respectively. Recombinant His-tagged RhoGDI and TbRhoGDI were expressed in *E. coli *M15 and BL21DE3, respectively, and purified from the soluble fraction of bacterial extracts on Ni^2+^-NTA agarose (Qiagen, Hilden, Germany). Membranes of insect or HeLa cells expressing Rho GTPases (1.0 mg of protein each) were rotated end-over-end for 15 h at 4°C in 200 μl of 20 mM Tris-HCl pH 7.5, 1 mM EDTA, 1 mM dithiotreitol and 0.1 mM phenylmethylsulfonyl fluoride containing 40 μM His-tagged RhoGDI. The samples were centrifuged at 100,000 × *g *for 10 min at 4°C and the supernatants used for Western analysis as described previously [23]. In these experiments His-tagged RhoGDI was always recovered in the supernatant.

### Plasmid construction and *Dictyostelium *transformation

To express green fluorescent protein (GFP) fused to the amino terminus of TbRhoGDI, bovine RhoGDI1 and human RhoGDI2 (LyGDI), cDNA fragments were amplified by PCR and subcloned in frame at its 5'end to the coding region of the red shifted S65T mutant of *Aequoria victoria *GFP in the transformation vector pDEX-GFP [59]. Plasmids were introduced in *Dictyostelium *GDI1^- ^cells by electroporation [60]. After selection for growth in the presence of G418 (Sigma, St. Luis, MO, USA), GFP-expressing transformants were confirmed by visual inspection under a fluorescence microscope. A GDI1^- ^strain re-expressing *D. discoideum *RhoGDI1 (GDI1) has been described previously [23].

### Heterologous complementation in yeast

For heterologous expression in *S. cerevisiae*, the yeastexpression plasmid pYES2 (Invitrogen Life Technology, San Diego, California) containing the URA3 auxotrophic marker was used to express Tb*gdi *under the inducible promoter GAL1. Tb*gdi *was excised from pGADT7-Tb*gdi *using EcoRI and XhoI and was directionally inserted into pYES2. INVsc1 yeast strain cells were grown to OD_600 _0.8 in YPD medium (Sigma Aldrich, St. Luis, Montana) at 30°C and then transformed with pYES2-Tb*gdi *by chemical transformation (Sigma Aldrich, St. Luis, Montana) according to the manufacturer's instructions. Transformants were selected on glucose – Ura medium plates. For expression, transformants were grown overnight in minimal medium lacking uracil, containing only raffinose as carbon source. After 24 h cells were switched to a medium containing raffinose and galactose in order to induce protein expression from the plasmid.

### Western blotting

*D. discoideum *cells were collected by centrifugation and pellets were lysed directly in Laemmli buffer. Proteins were separated by SDS-PAGE and transferred to nitrocellulose membranes. The membranes were incubated with *D. discoideum *GDI1 specific monoclonal antibody K8-322-2, or GFP specific monoclonal antibody K3-184-2 [23] followed by an anti-mouse peroxidase-conjugated antibody. GST was detected with a polyclonal antibody from Amersham (Buckinghamshire, UK). Immunoreactive bands were visualized by enhanced chemiluminescence (Amersham, Buckinghamshire, UK) following the procedures recommended by the manufacturer.

### Cell biology methods

To evaluate the distribution of the number of nuclei, *D. discoideum *cells were allowed to grow on coverslips, were then fixed in cold methanol (-20°C) and nuclei were stained with 4',6-diamidino-2-phenylindole (DAPI) (Sigma, St. Luis, Montana). Cells were visualized with a Leica DMR fluorescence and images were acquired with a Leica DC350FX camera (Leica, Wetzlar, Germany). Chemoattractant-induced F-actin formation in aggregation competent cells was determined through staining with TRITC-labeled phalloidin (Sigma, St. Luis, Montana) and quantitated as described previously [35] using a fluorimeter (Photon Technology Intl., Seefeld, Germany).

## Authors' contributions

MM performed yeast two hybrid analyses and complementation experiments with TbRhoGDI. GB performed cloning and sequence analysis. EP performed gene expression analysis. FR and AA conceived the study and drafted the manuscript. VS participated in the design of the study. FR also performed the translocation experiments and the complementation study with mammalian RhoGDIs and AA coordinated the work with *T. borchii*. All authors read and approved the final manuscript.
